# Connected attribute morphology for unified vegetation segmentation and classification in precision agriculture

**DOI:** 10.1016/j.compind.2018.02.003

**Published:** 2018-06

**Authors:** Petra Bosilj, Tom Duckett, Grzegorz Cielniak

**Affiliations:** Lincoln Centre for Autonomous Systems, School of Computer Science, University of Lincoln, UK

**Keywords:** Precision agriculture, Attribute morphology, Crop/weed discrimination, Max-tree

## Abstract

Discriminating value crops from weeds is an important task in precision agriculture. In this paper, we propose a novel image processing pipeline based on attribute morphology for both the segmentation and classification tasks. The commonly used approaches for vegetation segmentation often rely on thresholding techniques which reach their decisions globally. By contrast, the proposed method works with connected components obtained by image threshold decomposition, which are naturally nested in a hierarchical structure called the max-tree, and various attributes calculated from these regions. Image segmentation is performed by attribute filtering, preserving or discarding the regions based on their attribute value and allowing for the decision to be reached locally. This segmentation method naturally selects a collection of foreground regions rather than pixels, and the same data structure used for segmentation can be further reused to provide the features for classification, which is realised in our experiments by a support vector machine (SVM). We apply our methods to normalised difference vegetation index (NDVI) images, and demonstrate the performance of the pipeline on a dataset collected by the authors in an onion field, as well as a publicly available dataset for sugar beets. The results show that the proposed segmentation approach can segment the fine details of plant regions locally, in contrast to the state-of-the-art thresholding methods, while providing discriminative features which enable efficient and competitive classification rates for crop/weed discrimination.

## Highlights

• Segmentation and classification pipeline fully relying on attribute morphology.
• Strong locality of the approach avoids resulting foreground noise.
• Segmentation outputs regions directly, avoiding the component labelling step.
• Max-tree structure enables feature calculation during segmentation.
• Competitive classification of plant regions into crop/weed for varying plant types.

## 1 Introduction

Robust vision systems are a core technology for building autonomous robots in precision agriculture. Such systems automate time-consuming manual work in the field while increasing yield and reducing the reliance on herbicides and pesticides. To achieve this, the developed vision systems need to be able to monitor the crop and target only the specific plants that need treatment. Specifically, a number of approaches to discriminate value crops from weeds were developed [[1], [2], [3], [4]] and employed in robotic systems, which use this information to perform tasks such as mechanical weeding and selective crop spraying.

Mathematical morphology [5], and specifically attribute morphology, offers a versatile framework to perform multi-scale spatial analysis of image content in various image domains. Efficient implementations rely on hierarchical image representations and enable fast processing of large amounts of image data.

The contributions of this paper are threefold. Firstly, we propose a novel and unified pipeline for crop/weed detection and classification relying fully on attribute morphology. Secondly, we evaluate the approach on a publicly available sugar beets classification dataset [6] as well as a newly collected dataset focused on onion crops, which exhibits a higher variation in lighting and registration errors, thus requiring a more robust solution. Finally, we demonstrate the locality of the proposed approach and its ability to segment the fine details of plants, in contrast to the state-of-the-art global thresholding methods, as well as the discriminative properties of the provided features by obtaining competitive classification rates for crop/weed discrimination.

In the following section, we give a brief overview of related work from both precision agriculture and image morphology. Then, in Section 3 we explain the basic principles of attribute morphology, highlighting its advantages compared to standard structuring element morphology and explaining the data structure which enables the efficient implementation of the proposed pipeline. The core of the proposed approach is presented in Section 4. The data and experimental setup are explained in Section 5 followed by the results in Section 6. Finally, we conclude the paper and highlight future research directions in Section 7.

## 2 Related work

In order to apply per-plant treatments in precision agriculture, the vision system first performs segmentation, thus discarding all non-vegetation pixels, followed by classification of the remaining vegetation pixels to determine the correct treatment for plant regions of different types. We examine the related work through this two-step process.

Several choices for the segmentation step were explored in the literature, including colour-index based images calculated from *RGB* images (examples include *ExG* [7], *ExR* [8], *CIVE* [9], *VEG* [10]), normalised difference vegetation index (NDVI) images obtained from multi-spectral cameras as the difference-sum ratio of the near infra-red and visible red components [[11], [12]], images in colour spaces such as LAB [13] and different hue-based colour spaces [14]. The choice of input image is then thresholded to separate soil from vegetation. The threshold decision is usually reached globally, e.g. using Otsu's threshold selection method [15], resulting in methods sensitive to varying lighting conditions and requiring post-processing to locally adjust the output of thresholding by removing noise. More robust segmentation approaches were developed using machine learning-based methods [[16], [14], [2], [13]] but they come at an increased computational cost and are not well suited for real-time applications. For a recent overview of segmentation techniques applied to vegetation segmentation the reader is referred to [17].

Following the segmentation step, the foreground (vegetation) pixels are further classified into crops and weeds. Distinguishing between multiple weed classes is sometimes also of interest. Classifying only the vegetation pixels instead of all image pixels significantly reduces the computational load of classification. Colour information is often not enough to perform classification successfully, so additional information about texture and shape is often introduced. The two main approaches to classification are local pixel or grid-based approaches [[3], [18], [4]] and region-based approaches [[1], [19]], which can also be used in conjunction [12] to benefit from the advantages of both approaches. While the region-based approaches are typically very fast, as they deal with several tens of regions per image, they cannot cope well with occlusions to reach a fine-grained decision on a vegetation patch with overlapping crops and weeds. An additional component labelling step on the segmented image is required to prepare the input for a region-based classifier. On the other hand, pixel-based approaches suffer from high computational cost. This is partially mitigated by classifying only certain pixels on a grid and interpolating the classification values of other pixels. However, due to their high classification accuracy and robustness to partial occlusion, the strength of these approaches lies in applying them to the limited amount of pixels for which the region-based approaches do not reach a certain decision. In this paper, we propose a novel pipeline for both segmentation and region-based classification of plants, while the development of a complementary pixel-based classified is left for future work.

Mathematical morphology, with the recent developments in hierarchical image representation and attribute morphology, offers a versatile and efficient framework to perform multi-scale spatial analysis of image content in various image domains. Historically applied to segmentation problems [[20], [21], [22]], various morphological techniques were recently successfully applied to a large number of image processing and computer vision problems including object detection [[23], [24]], segmentation [[25], [26]], image retrieval [[27], [28], [29]], scene classification for remote sensing [[30], [31], [32]] and more. Fast processing is achieved by using a hierarchical image decomposition such as the max-tree [22], relying on efficient construction algorithms, parallelisation and simultaneous calculation of attributes used throughout the processing pipeline, allowing attribute morphology approaches to be applied to images as large as several Gpx, with reported speeds of up to 370 Mpx/s when using parallelisation [33].

## 3 Attribute morphology and hierarchies

Classical approaches to mathematical morphology rely on the concept of a structuring element (*SE*) to define the basic operations of erosion and dilation, and then opening and closing. The erosion operation will erode or shrink the boundaries of foreground regions, thus making the foreground shrink in size and removing all small foreground components, with dilation being the complementary operation. Combining erosion and dilation sequentially produces the opening operator, which enables the removal of small foreground components without introducing big changes to other foreground elements. The complementary operator of closing is obtained by first applying dilation and then erosion. The SE is a (typically small) binary image with a defined origin, with which the input image is “probed” to calculate the output image. Thus, an erosion corresponds to placing the SE at all positions in the input image, and placing a foreground pixel at the SE origin in the output image if all the SE pixels fall onto foreground pixels of the input image. Similarly, with dilation a foreground pixel is placed at the SE origin in the output image if any of the SE pixels fall onto the foreground in the input image. Finally, an opening operator corresponds to placing an SE at all positions in the input image, and placing foreground pixels on all the SE pixels in the output only if the whole SE falls into the foreground.

However, relying on a structuring element to define an opening has several drawbacks: the boundaries are not faithfully preserved, the method is not rotationally invariant (i.e. designing a single SE to respond to elongated objects is not possible and thus multiple line-like SEs with different orientations need to be used), and shape and size are treated together, making it difficult to filter objects based on only one of these characteristics.

**Fig. 1:**
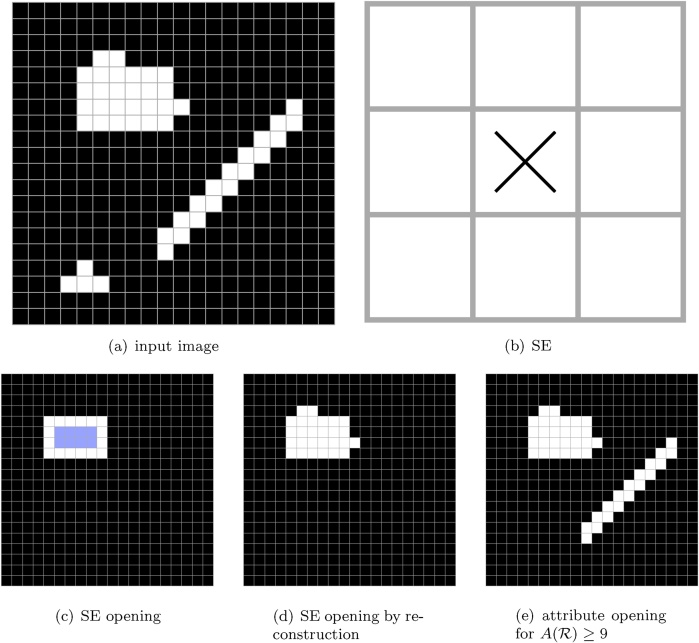
An example binary image is shown in (a), and the goal is to discard the objects smaller in size than 9 pixels. In SE morphology, an SE such as the one in (b) would be used (SE origin marked with ×). The result of an opening of the image (a) with the SE in (b) is shown in (c). As the opening is made by chaining an erosion and a dilation operation with the same SE on the image, the result of the erosion is also shown shaded on (c). The subsequent dilation and the resulting opening do not retrieve the fine details of the region edges, nor the elongated object. Opening by reconstruction using the same SE is shown in (d), where the fine details of the larger object are retrieved, but the thin object is still missing. Finally, in (e) we show an attribute opening (more specifically: area opening), where no SE is used. Instead, the regions are kept based on a criterion “is the area of the region larger than 9”, thus correctly capturing both the large rectangular and the thin elongated region, and discarding the smallest region in the example.

To address these problems, morphology has moved in the direction of connected filters [[34], [26]]. The first such operators were binary opening and closing by reconstruction [[35], [36]], which still rely on a SE to define which foreground regions should be removed from the image but fully reconstruct all the remaining components. The problems of rotational invariance and decoupling of shape and size are addressed in attribute morphology [[37], [22]], in which the SE is omitted completely. Instead, in attribute morphology the decision to keep or discard is reached at region level, thus only keeping the regions where an attribute satisfies a certain criterion. This allows using criteria such as “area of the region is greater than 100” to process the input image. The difference between an opening with an SE, opening by reconstruction and an area opening on a binary image is shown in Fig. 1.

All the basic operators defined in SE morphology are *increasing*, meaning that they preserve the order of binary images such that *B*_1_ ⊆ *B*_2_ then *F*(*B*_1_) ⊆ *F*(*B*_2_) where *F*(·) is the operation of erosion, dilation, opening or closing by an SE. This allows the extension of binary SE morphology to greyscale images relying on the principles of threshold decomposition [38] and stacking [39]. A greyscale image $f:E\to \mathbb{Z},E\subseteq {\mathbb{Z}}^{2}$ is represented by its upper-level sets, defined as $L^{k}=\{f\geq k\}$ with k∈ℤ, i.e. the set of images obtained by thresholding an image at all possible values of their pixels (similarly one can work with lower-level sets $L_{t}$). The result of applying a morphological operator to a greyscale image can then be expressed by applying the binary operator to each level set of the image. Attribute morphology can similarly be extended to greyscale images if increasing attributes are used. Removing regions based on their size attributes (e.g. area, diameter of smallest enclosing circle, span along an axis, etc.) leads to increasing operators, called *attribute openings*. Attribute filters relying on non-increasing attributes with a different invariance property (e.g. shape filters produced by scale-invariant attributes such as Hu's moments [40]) were also developed and formalised [41].

**Fig. 2:**
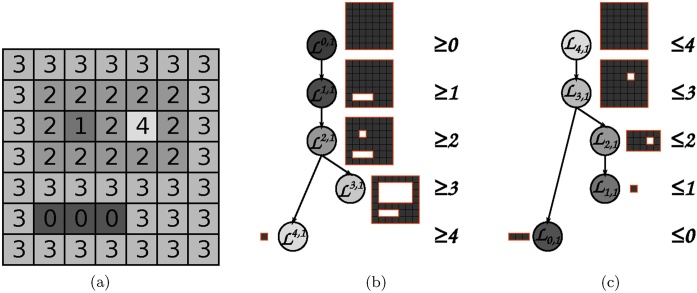
The max-tree for the shape in (a) is displayed in (b), and the min-tree in (c). The regions corresponding to the nodes of the trees are displayed besides them, with the level sets indicated inside the nodes.

In order to efficiently apply attribute morphology operators to a greyscale image, the image is represented as a max-tree [22]. The max-tree is a hierarchical image decomposition based on the threshold decomposition of the image into its upper-level sets. Each upper level set $L^{k}$ is composed of its peak components $L^{k,i}$ (*i* from some index set), comprising the connected components (regions) of maximal extent, and these peak components are nested for decreasing values of *k*. The max-tree structure thus has local image maxima in the leaves and is well suited for processing bright image details. Traversing a single branch of the tree from the leaf to the root corresponds to going through all the foreground regions obtained by thresholding the image around the local maximum with a decreasing threshold. Its dual, the min-tree, is constructed from lower-level sets $L_{k}$ and is suited for processing dark image structures. The regions of the max-tree (resp. min-tree) correspond to the maximal (resp. minimal) extremal regions used as candidates for the maximally stable extremal regions (MSER) detector [42] and hierarchies can be used for an efficient implementation of this detector [27]. The response of the MSER detector comprises regions stable through a range of threshold values for the given image. Both hierarchies are shown in Fig. 2. Different hierarchies also exist allowing interaction with both bright and dark image structures simultaneously (see e.g. [26] for an overview).

## 4 Method

In this section, we explain in detail the proposed pipeline for crop/weed detection and classification based on attribute morphology. We also give a short overview of the global thresholding methods to which the proposed vegetation segmentation technique is compared.

In brief, each image is represented by the max-tree hierarchy [22], a single structure on which all the processing is performed. The segmentation step selects regions from the hierarchy based on their size stability over a series of grey levels, simultaneously calculating several attributes for the selected regions. This enables reaching a decision about each region locally, in contrast to the global thresholding operations typically employed for this task. The output of the segmentation process is a set of distinct regions with associated attributes, which are directly used as region features for the classification scheme. This enforces greater coherence between the segmentation and classification steps, and partially eliminates the need for pre-processing. Additionally, a number of operations typically associated with pre- or post-processing can be performed simultaneously with the segmentation task.

### 4.1 Global thresholding

In order to obtain a baseline segmentation performance and provide a fair comparison of the proposed segmentation approach, we examine the segmentation performance of two different global thresholding algorithms.

*Otsu's thresholding method* [15] is a very popular choice for vegetation segmentation [[43], [44], [3]]. This method relies on the histogram of the distribution of the pixel values present in the image to examine all possible thresholds and propose the one resulting in the greatest inter-class variance between the background and foreground classes. Consequently, the method does not take spatial information into account and often results in small foreground noise.

We also compare our approach to the *Robust Automatic Threshold Selector (RATS)* algorithm [45], as it can adapt to image content (as demonstrated for example on segmenting blood vessels [46], whose filament-like structure resembles some of the plant regions in our target application). The method does not rely on the image histogram. Instead, the threshold *T* for the image is determined as the average of image intensities weighted by the image gradient (allowing different gradient operators), where only the pixel values with gradient values above a certain noise level *ηλ* are taken into account:

(1) $$T=\frac{\sum_{(x,y)\in E}w(x,y)f(x,y)}{\sum_{(x,y)\in E}w(x,y)},$$

$$w(x,y)=\left\{\begin{array}{cc} G(x,y), & \mathrm{if}\;G(x,y)>\eta \lambda ,\\ 0, & \mathrm{otherwise}, \end{array}\right)$$

where *G*(·) is the image gradient at pixel position (*x*, *y*), *λ* is the adjustable sensitivity parameter of the method dependant on the gradient measure used (we use *λ* = 3 and the Sobel operator, according to [47]), and *η* is used to control sensitivity to background noise.

The methods for automatic threshold selection such as Otsu's method and RATS, which reach their decision globally, often exhibit poor performance under uneven lighting conditions, in the presence of noise or a highly-featured background, and require a separate post-processing steps to remove small isolated responses. They also require balanced classes or a minimum level of image content to produce good results.

### 4.2 Vegetation segmentation

The main motivation for the proposed approach is the locality of the processing techniques based on the max-tree hierarchy in order to overcome the drawbacks of the global thresholding methods. Even in the presence of noise (cf. Figs. 7(e) and (f) and 5(c)), the plant regions are visually easily distinguishable due to their high local dissimilarity with their background. For this reason, we chose the max-tree as the underlying data structure in the presented pipeline, as it allows to work with the bright image regions and examine them while taking into account only their local neighbourhood.

**Fig. 3:**
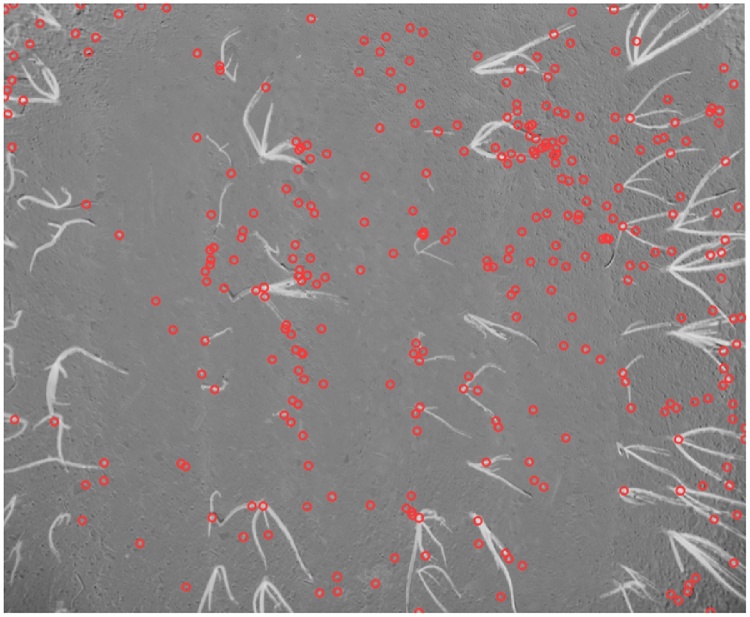
A selection of image maxima on an NDVI image (corresponding to max-tree leaves) with extinction values ≤30. Note that we show here a more constrained set than the one used in the algorithm for visualisation purposes.

The local maxima contained in the max-tree leaves can serve as a good set of initial markers when looking for plant segments (and more generally, in object detection tasks). The number of local maxima per image can be very large due to noisy images as well as registration errors (e.g. in the order of 300,000 for the images in our onions dataset). To further reduce the search space, we order the local maxima according to their *extinction values* [48]. Any increasing attribute can be used to calculate this contrast measure, which corresponds to the maximal size of the attribute filter such that the local extremum is not affected by filtering. We chose to work with extinction values based on the grey-level range attribute which measure the contrast from the background corrected for noise. All the local maxima with extinction values smaller than 10 (in the range of 1–255) were discarded, keeping only about 3% of the initial markers as candidate maxima for the rest of the pipeline. A selection of max-tree leaves by extinction value is shown in Fig. 3.

**Fig. 4:**
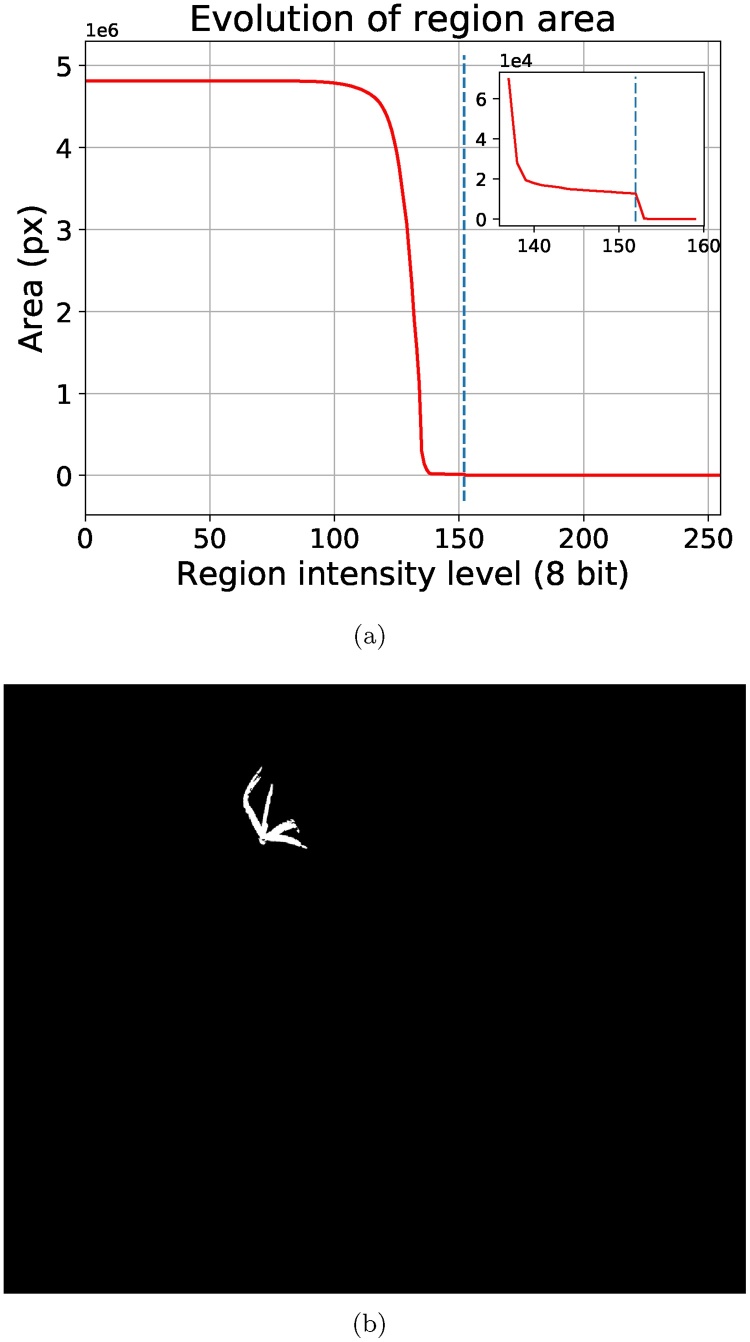
Evolution of the region size following a branch of the max-tree from the leaf to the root is shown in (a), corresponding to thresholding with a decreasing threshold (the leaf region corresponds to the highest threshold). The largest growth over *Δ* = 30 grey levels is detected for *T* = 104 (indicated with a vertical blue line), and the detected region corresponding to that threshold is shown in (b). (For interpretation of the references to colour in this figure legend, the reader is referred to the web version of this article.)

The core step of the segmentation algorithm relies on a similar assumption to the MSER region detector [42] and the ultimate opening filter [49]: due to the good local contrast of the foreground (plant) regions, the area of the upper-level set containing a local maximum $p_{\max}\in L^{k,i}$ will be stable through a range of grey levels *k* and will then rapidly increase over a small number of grey levels. This rapid growth of the region is a good indicator of the merging between the contrasted foreground object and the background. We examine the rate of growth for all the regions along the max-tree branches associated with the local maxima kept after extinction value filtering. A stability parameter *Δ* is introduced, and at each node the growth factor *G*(·) over a span of *Δ* grey levels is calculated as:

(2) $$G(R_{k})=\frac{A(R_{k+\Delta })-A(R_{k})}{A(R_{k})},$$

where $R_{k}$ is the upper level set $L^{k,i}$ of the current branch at grey level *k*, and *A*(·) stands for the area of the region. In principle, we are interested in the region with the largest growth factor along each selected max-tree branch. An example of the evolution of the area attribute along a branch of a max-tree is shown in Fig. 4.

In practice, we noticed that large growth values can be obtained from regions with small area, since adding a few pixels to the region could easily result in the region size increasing several times. However, as the growth is calculated discretely and with a fixed value of *Δ*, several regions of similar size and with a similar growth factor usually exist along a single tree branch where a plant region is present in the image, which we can use as an additional indicator when selecting regions. Thus, after selecting the region B with the largest growth factor G(B) along the tree branch as the best candidate, we adjust our choice to favour slightly larger and repeated regions with the following steps:

(1) examine the regions R with G(R)<G(B) and $G_{\min}<G(R)$ in order of descending growth,
(2) find the one with the largest growth factor such that A(R)>KA(B),
(3) keep examining the regions $R^{\prime }$ in the order of descending growth and count the number *l*_similar_ of consecutive regions $R^{\prime }$ s.t. $\left(1-e_{A})A(R)\leq A(R^{\prime })\leq (1+e_{A})A(R\right)$,
(4) if *l*_similar_ ≥ *l*, assign the region R to B as the best candidate and repeat step (1).

In step 1, all regions above the minimal allowed growth *G*_min_ are examined in order of descending growth. Then, step 2 ensures that the newly considered candidate region R is sufficiently larger than the previously selected region B, controlled by the parameter *K*. Step 3 examines the regions of similar growth and counts the ones with similar area (controlled by *e*_*A*_) to the considered region R. If *l* such regions are found, the considered region R is assigned as the best region B in step 4. After this adjustment, the candidate region B is accepted as a foreground region.

At the end of the selection process for each branch, the selected region can additionally be compared to an area threshold *T* and discarded if it is too small regardless of its growth factor. This filtering step introduces no additional complexity to the approach, takes the place of costly post-processing steps for noise removal, and further can be implemented using any increasing attribute other than area describing the region size.

The output of this segmentation step is a list of accepted regions which can be further processed on the max-tree structure, so that no component labelling step is required to prepare the segmentation output for classification. While exact duplicates are discarded from the list of accepted regions, all other nested regions are returned and no additional similarity filtering is performed as in MSER [42], resulting in detection of near-duplicated regions. This is a possible point of improvement in the approach, however care should be taken in the way that nested regions are handled so that no useful information is discarded. For instance, while some of the nested regions will correspond to the same plant with only a few pixels difference in the region selection, which can be discarded; in other cases (e.g. overlapping vegetation) the nested regions will represent sub-structures of the largest segmented region, which should be kept.

### 4.3 Plant classification

In order to perform classification of the selected regions, several attributes were calculated for each region and concatenated into a feature vector describing that region. While any attribute can be calculated if the coordinates and values of all the pixels belonging to a region are known, attribute morphology has a preference for certain types of attributes which can be computed efficiently. Efficiently here means that the attribute value of a region can be calculated from the attribute values of its subregions (or a small number of auxiliary stored values) and examining only pixels which are unique to that region. For instance, the area of a region can be expressed as the sum of areas of all its subregions plus the number of unique pixels of that region. This formulation allows for an efficient computation on the max-tree as the attribute values can be propagated from the leaves towards the root of the tree and re-used when needed, ensuring that even if the attribute is calculated for every region present in the hierarchy, no image element is examined more than once. We consider the following four shape features and one statistical feature:

• *Solidity* is defined as the ratio of the region area to the area of the convex hull of the region [3]. Only the pixels on the hull are propagated through the tree and used when determining the convex hull of the parent region.
• *Eccentricity* of a region measures how much a conic section differs from being a circle. It is calculated as the eccentricity of the equivalent ellipse positioned at the centre of mass of the region with the same second order moments as the original region. The eccentricity of an ellipse is defined as $\sqrt{1-\frac{b^{2}}{a^{2}}}$, where *a* and *b* are the lengths of the major and minor semi-axes respectively. It can be expressed and calculated from centralised image moments.
• *Circularity* measures a similar property and is typically defined as $\frac{A(R)}{P{\left(R\right)}^{2}}$ where *P*(·) is the perimeter of the region. However, as the perimeter is not an increasing attribute nor can it be computed incrementally on a hierarchy of nested regions, we use a moment-based measure of circularity [50]:
  $\frac{A(R)}{2\pi \mu_{2,0}(R)\mu_{0,2}(R)}$, where *μ*_*p*,*q*_(·) are the centralised moments for the associated region.
• *Non-compactness* is an elongation measure of the region, and corresponds to the first moment invariant of Hu [40] corrected for application on the discrete image space [25]. It is defined as $2\pi (\frac{I(R)}{A{\left(R\right)}^{2}}+\frac{1}{6A(R)}).$
• *Range* is a statistical feature measuring the span of grey levels included in the region on the NDVI image, $\left|f_{\max}(R)-f_{\min}(R)\right|$.

These features are concatenated into a feature vector for every region, and every feature is normalised across the dataset. The normalised feature vectors associated with the regions are used as input to train and test the classifier. While the advantage of the region-based approach is that it only classifies a few objects per image, it cannot accurately classify the pixels of regions which contain both value crop and weed pixels. Such regions are assigned to the “mixed” vegetation class, and a pixel-based classifier such as in [12] would need to be added to the pipeline to handle these cases. The classification step is realised by a support vector machine (SVM) [51], as a two-class classification problem with rejection based on classification confidence *c*. Each region is classified as either crop or weed, or rejected if the classification confidence is below *c*%, a parameter which can used to minimise false negatives or false positives.

## 5 Data and experimental setup

### 5.1 Image acquisition

Our approach is based on multi-spectral sensing of vegetation, combining standard three-colour RGB and an additional near infra-red (NIR) channel into a 4-channel RGBN image. The combination of visible (i.e. red channel) and NIR information is a good indicator of photosynthetic plant activity [11] and hence allows for segmentation of vegetation from the background. In particular, we employ the normalised difference vegetation index (NDVI), which can be calculated as the normalised difference between visible and near infra-red channels:

(3) $$\mathrm{NDVI}=\frac{\mathrm{NIR}-\mathrm{VIS}}{\mathrm{NIR}+\mathrm{VIS}}.$$

We validate the performance of our system on two distinctive sources of NDVI information: a publicly available dataset of RGBN images [6] collected under controlled lighting conditions, and our own dataset of onions collected under natural light variation. The acquisition procedure as well as the way ground truth was obtained for selected tasks is detailed for each dataset.

**Fig. 5:**
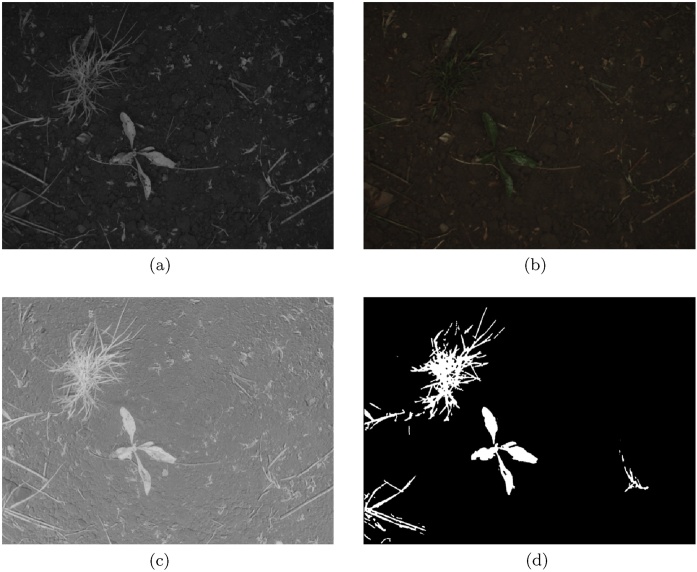
Examples of NIR and RGB images from the Sugar Beets 2016 dataset are shown in (a) and (b) respectively. The resulting NDVI image is shown in (c), while the ground truth is displayed in (d).

The *Sugar Beets 2016* dataset contains RGBN images of sugar beet fields collected with a multi-spectral camera mounted on an agricultural robot BoniRob [6]. The camera model JAI 130-GE uses a splitting prism optically separating near infra-red and visible spectra, which are then captured by two separate image sensors resulting in 4 perfectly aligned RGBN channels. The camera was mounted at a height of 85 cm above the ground and provides images of 1296 × 966 pixels, which together with the employed optics results in 3 px/mm resolution and a field of view corresponding to a rectangular 24 cm × 31 cm patch of ground. The lighting conditions in this system were tightly controlled by placing the camera in a dedicated shroud, protecting it from the sunlight and employing artificial lights for constant illumination. An example image from the dataset is shown in Fig. 5.

**Fig. 6:**
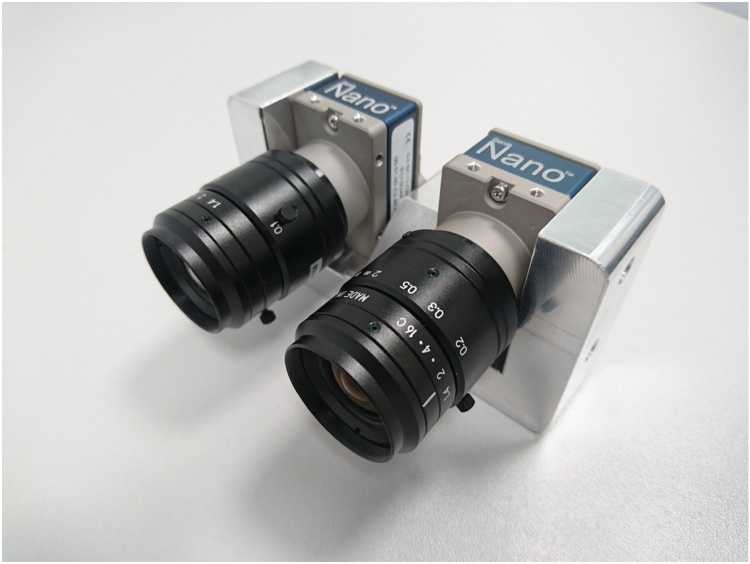
Dual-camera NIR + RGB setup with Teledyne DALSA Genie Nano cameras.

**Fig. 7:**
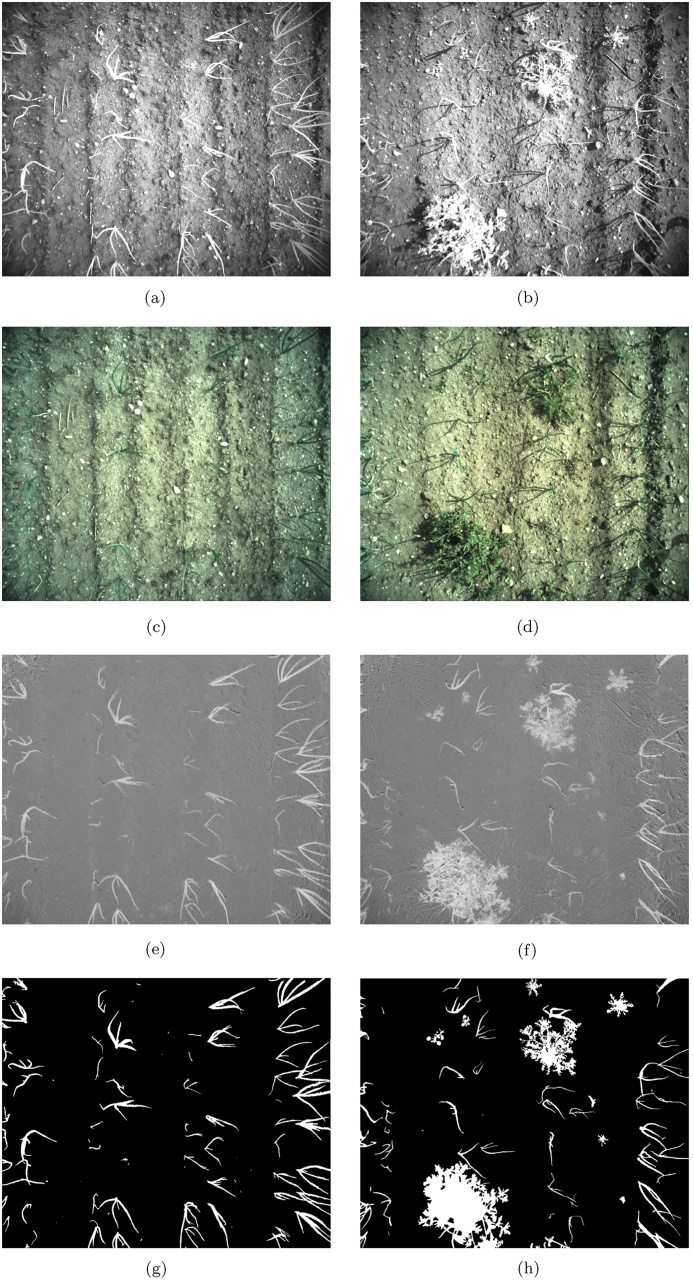
The original NIR images are shown in (a,b) with the registered RGB images in (c,d). The resulting NDVI images are displayed in (e,f) with the corresponding manually segmented ground truth images shown in (g,h).

The *Onions 2017* dataset was collected by the authors during multiple sessions at fields in Lincolnshire, UK. The dataset was acquired using a two-camera setup with RGB and NIR cameras (Teledyne DALSA Genie Nano) mounted 5 cm apart (setup shown in Fig. 6) and deployed on a manually pulled cart over the bed of onion plants. In this case, the optical centres of the RGB and NIR sensors were misaligned and therefore the images required an additional registration step. The resulting RGBN image is of high resolution (2464 px × 2056 px) but can exhibit parallax errors, especially with high and mature plants. In our case, the camera was mounted at a height of around 100 cm above the ground, which together with the employed optics resulted in 2.5 px/mm resolution covering a 100 cm × 85 cm patch of ground. Additionally, we did not use any lighting control mechanisms and relied only on the automated gain control built into the cameras to alleviate uneven lighting conditions. Examples of the resulting images are shown in Fig. 7.

**Fig. 8:**
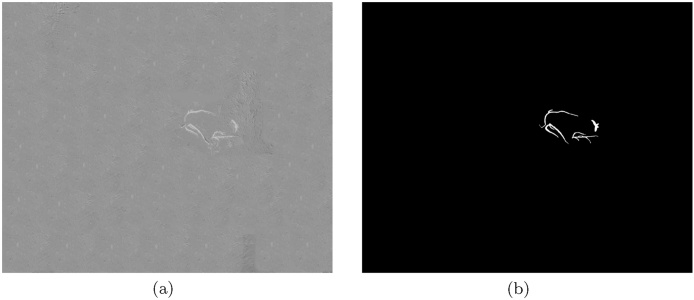
The artificially constructed low-vegetation NDVI image is shown in (a) (based on the original NDVI image shown in Fig. 7(f)). The corresponding ground truth is shown in (b).

The *LowVeg* dataset comprises a single artificially constructed low-content onion NDVI image, containing only 4 vegetation regions in close proximity. This image was constructed from the NDVI image shown in Fig. 7(f) by selecting a large portion of the ground from the same image and masking all other vegetation in the image with the ground patch. The resulting NDVI image looks realistic due to the consistent texture, and is shown, with the corresponding ground truth, in Fig. 8. This image was used to test the behaviour of the proposed approach in images with low vegetation content.

### 5.2 Data annotation

The *Sugar Beets 2016* dataset provides per-pixel segmentation and classification results for selected images. In order to obtain the ground truth for the segmentation task, selected images from the *Onions 2017* were manually segmented by first selecting a threshold giving visually good results, and then adjusting the thresholding results on a by-pixel basis based on the NDVI and RGB images.

In order to evaluate the performance of the region classifier, per-region ground truth data are required. This was obtained by manually assigning classes to each region produced by our segmentation step. We also observed that while labelling the segments, approximately 40% of the regions returned by the segmentation are near-duplicates. The images from the *Onions 2017* dataset were classified based on the visual appearance of the segmented regions in the NDVI and RGB images, while ambiguous and small regions (mostly located near the corners of the images) present in the segmentation results were discarded. Images from the *Sugar Beets 2016* dataset were also manually labelled for the purpose of the experiments, based on the ground truth images provided and aided by the visual appearance of the segmented regions in the NDVI and RGB image. While the original ground truth is provided per-pixel, we manually classify the segmented regions as ‘mixed’ where appropriate and do not distinguish between different types of weeds. The subsets of the datasets used for segmentation and classification tasks are detailed in Table 1.

**Table 1:** Details of the datasets used for evaluating the proposed approach. In the class distribution column, ‘V’ stands for ‘vegetation’, ‘B’ for ’background’ (soil), ‘C’ for ‘crop’, ‘W’ for ’weeds’ and ‘M’ for the ‘mixed’ class.

Dataset	#img	Ground truth	#samples /img	Class distribution
*Segmentation*				
*Onions 2017*	4	Manual	2422 px × 1988 px	V – 7.10% B – 92.90%
*LowVeg*	1	Manual	2422 px × 1988 px	V – 0.25% B – 99.75%
*Sugar Beets 2016*	282	Provided	1296 px × 966 px	V – 6.08% B – 93.92%
*Classification*				
*Onions 2017*	40	Labelled seg. output	224 regions (average)	C – 64% W – 26% M – 10%
*Sugar Beets 2016*	20	Labelled seg. output	37 regions (average)	C – 24% W – 69% M – 7%

### 5.3 Performance evaluation

We evaluate the performance of classification and segmentation on the presented datasets in terms of precision, recall and *F*_1_ measure. Precision indicates the chance that a selected positive sample is correct, and is calculated as:

(4) $$p=\frac{\mathrm{TP}}{\mathrm{TP}+\mathrm{FP}}.$$

Recall, or specificity, refers to the percentage of relevant samples that were selected, expressed as:

(5) $$r=\frac{\mathrm{TP}}{\mathrm{TP}+\mathrm{FN}}.$$

In both Eqs. (4) and (5), TP and FN stand for the number of true positive and false negative samples. For the purpose of precision-recall curves, calculate the interpolated precision at a recall level *r*, expressed as the highest achieved precision at any recall level *r*′ > *r*, which is done to avoid the distinct saw-tooth shape of precision recall curves [52].

The *F*_1_-measure is a singular quality measure calculated as the harmonic mean of precision and recall:

(6) $$F_{1}=\frac{2\mathrm{pr}}{p+r}.$$

It is often a more faithful estimator of quality than simple accuracy, as it takes into account the imbalance of the classes [52]. Confusion matrices and other performance measures can be calculated from precision and recall, given sample size and class distribution (cf. Table 1).

### 5.4 Experimental setup

For the purpose of the **segmentation** experiments, the parameters for steps 1–4 of the algorithm were determined empirically and set as follows: minimal allowed growth *G*_min_ = 10, size difference factor *K* = 15, area difference *e*_*A*_ = 0.15, and minimal limit on the number of similar consecutive regions *l* = 6. The behaviour of the proposed segmentation algorithm is analysed under varying values of the stability parameter *Δ*.

In the **classification** experiments, we divide the dataset images into *k* = 10 equal subsets. The SVM is then trained on *k* − 1 subsets, while the remaining subset is used for performance evaluation. As the classifier treats each region as a separate sample, this differs from a typical *k*-fold cross validation. We chose to divide the set of input images rather than the set of input regions into *k* subsets, to ensure that no sample from the testing set is used in training the classifier. For training, all the samples from the selected images are further divided into a training set consisting of 70% of the samples, while the remainder is used as a validation set. Different SVM kernels and parameters are compared on the validation set (cf. Table 2), and the results are reported for the set of parameters performing best on the validation dataset for each experiment. Since we train a two-class classifier, the samples of the mixed class are presented to the classifier twice: once as ‘crop’ samples and once as ‘weed’ samples. Additionally, the smaller of the two classes is increased to the size of the other class by repeating random samples from the larger class, which achieves a balance between classes on the samples of the training set. The SVM classifier returns the probability *p* that a sample belongs to a class, where *p*_crop_ = 1 − *p*_weed_.

**Table 2:** Support vector machine parameters used in the experiments.

Kernel type	Parameters
Linear	*C* ∈ {1, 10, 100, 1000}
RBF	*C* ∈ {1, 10, 100, 1000} *γ* ∈ {0.001, 0.0001}
Polynomial	degree ∈{2, 3, 4}

## 6 Experimental results

### 6.1 Vegetation segmentation

**Fig. 9:**
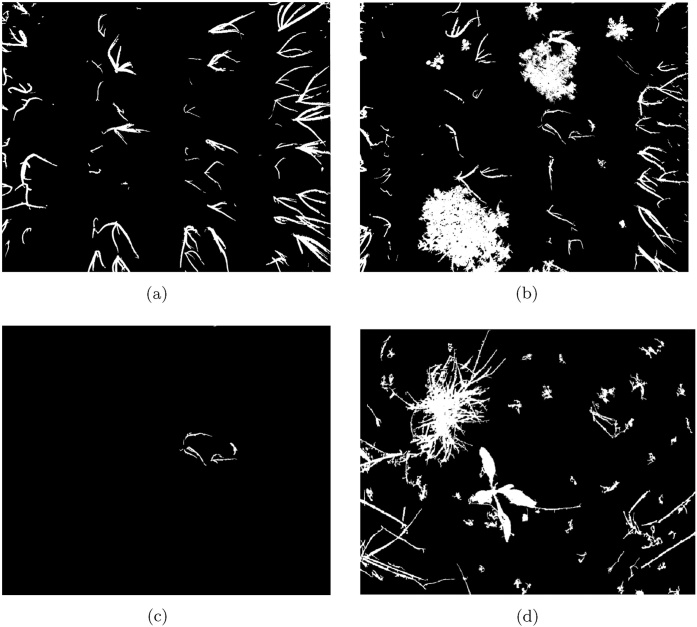
The results of the proposed max-tree based segmentation on the NDVI images in Fig. 7(e) and (f) are shown in (a,b). For the artificial NDVI example in Fig. 8(a) the segmentation result is shown in (c), and for the selected sugar beets image from Fig. 5(c) in (d).

**Fig. 10:**
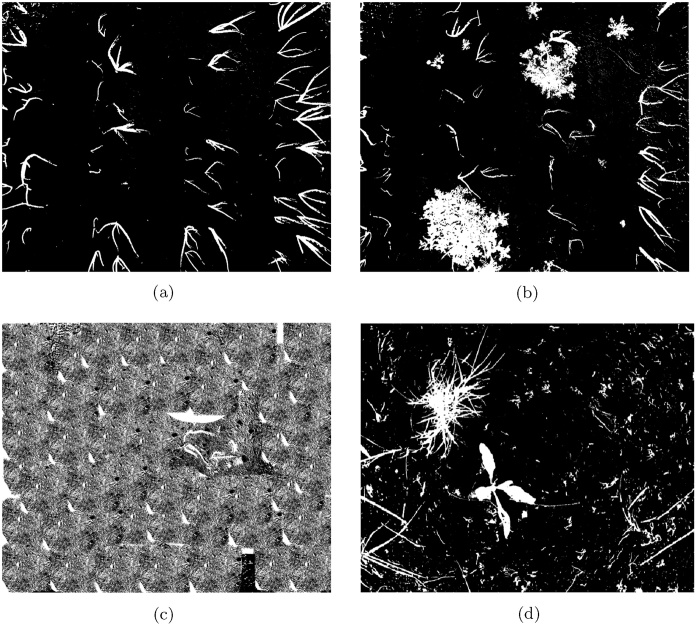
The results of the Otsu's thresholding method on the NDVI images in Fig. 7(e) and (f) are shown in (a,b). For the artificial NDVI example in Fig. 8(a) the segmentation result is shown in (c), and for the selected sugar beets image from Fig. 5(c) in (d).

**Fig. 11:**
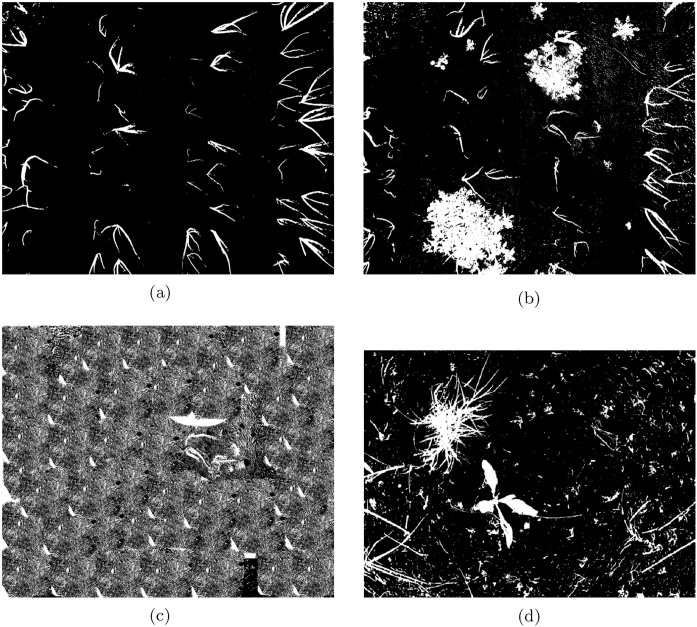
The results of RATS algorithm on the NDVI images in Fig. 7(e) and (f) are shown in (a),(b). For the artificial NDVI example in Fig. 8(a) the segmentation result is shown in (c), and for the selected sugar beets image from Fig. 11(d) in (d).

We first visually compare the outputs of the segmentation tasks, calculated for the dataset subsets detailed in Table 1. The output of the proposed method is shown in Fig. 9, Otsu's thresholding method in Fig. 10 and RATS in Fig. 11, allowing a qualitative analysis of the results. The false positives in both global thresholding approaches come from the small foreground noise caused by the soil not being particularly uniform, but are avoided by the max-tree approach. While removing noisy responses only a few pixels in size requires a costly post-processing operation following global thresholding, a minimal size criterion can be included in the max-tree segmentation approach (cf. Section 4). Relying on the max-tree, the false positives come partially from imprecise plant borders (some in the ground truth and some in the segmentation output), and partially from detecting strong structures present in the background but not present in the ground truth (e.g. in the sugar beet image, cf. Fig. 5, Fig. 9).

Moreover, in the near-absence of vegetation content as in Otsu's thresholding and RATS (shown in Fig. 10(c) and 11(c) respectively), both global approaches segment the background textures as foreground structures. This is due to the Otsu's thresholding expecting roughly balanced classes, and low-content images can cause the RATS threshold to not be well-defined.

**Fig. 12:**
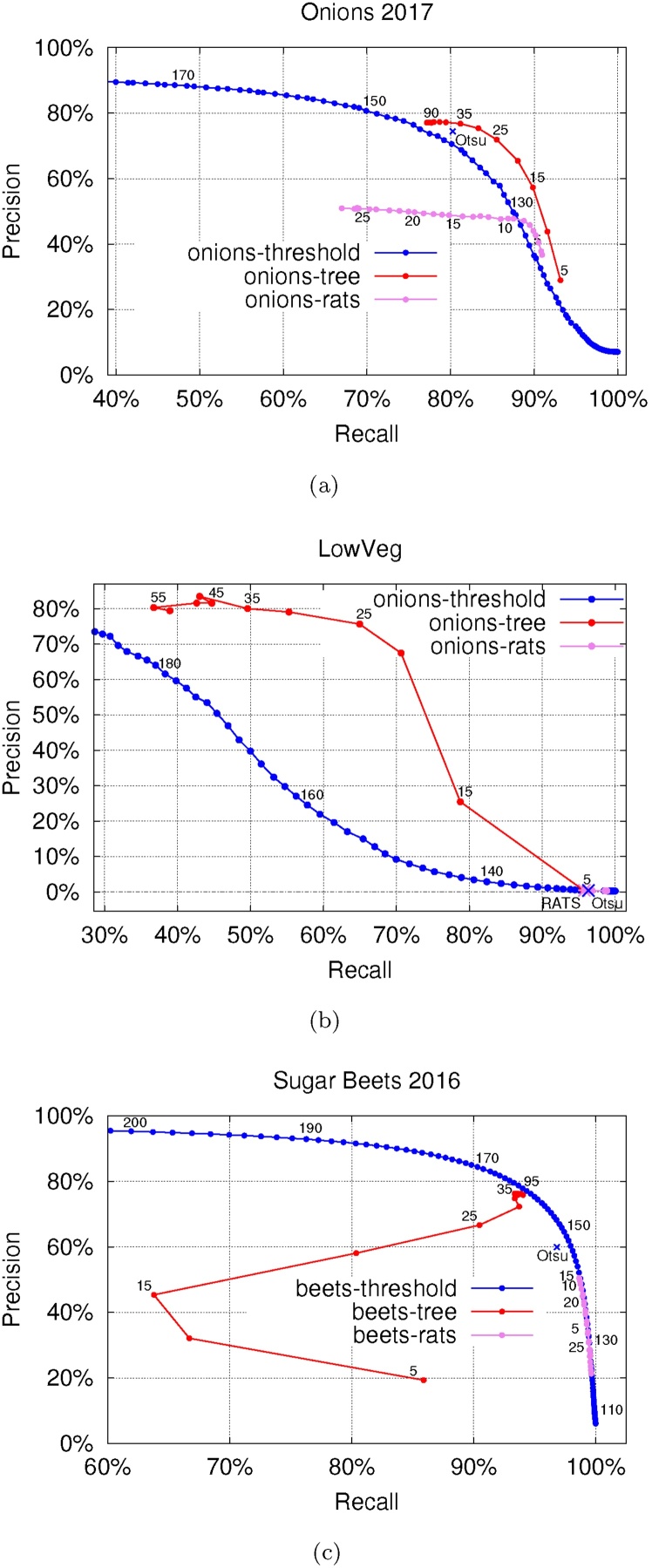
Precision-recall curves for the (a) *Onions 2017*, (b) *LowVeg* and (c) *Sugar Beets 2016* dataset. The blue line corresponds to varying the threshold from 0 to maximal pixel intensity (averaged per image). As Otsu's algorithm does not return the same threshold for every image, the resulting performance does not coincide with any point on that line.

The performance of the methods is compared in Table 3, with the precision-recall curves shown in Fig. 12. Our method achieves the highest *F*_1_ measure on all the datasets. While all the approaches have a high recall score across all the datasets, the high *F*_1_ measure of the proposed method comes from achieving high precision. This is especially pronounced on the *LowVeg* dataset which is successfully segmented by the max-tree approach, while both other methods achieve an *F*_1_ score below 1%. Additionally, while the best value of the *Δ* parameter listed differs significantly between the onions and sugar beets datasets, we note that the performance is stable for *Δ* ∈ {20, 30, …, 55} for all datasets (*F*_1_ measure difference within 3% on *Onions 2017*, 15% on *LowVeg*, 2% for *Sugar Beets 2016*). For the sugar beets, the performance was stable for an even greater range up to *Δ* = 95, which is more than a third of the pixel intensity range.

**Table 3:** Segmentation performance for different approaches. ‘Range’ signifies that the method achieves the same performance for a range of parameters.

Method	Otsu	RATS	max-tree
Dataset	*Onions 2017*		
Precision	74.41%	47.78%	**75.36%**
Recall	80.25%	**87.54%**	83.32%
*F*_1_	77.22%	61.82%	**79.14%**
Parameters	–	*η* = 8	*Δ* = 30
Dataset	*LowVeg*		
Precision	0.40%	0.44%	**75.66%**
Recall	**96.33%**	95.77%	64.96%
*F*_1_	0.80%	0.88%	**69.90%**
Parameters	–	range	*Δ* = 25
Dataset	*Sugar Beets 2016*		
Precision	59.93%	50.52%	**76.21%**
Recall	96.81%	**98.64%**	93.87%
*F*_1_	74.03%	66.82%	**84.13%**
Parameters	–	*η* = 14	*Δ* = 45

The best performance in terms of precision, recall and F_1_ measure for each dataset is highlighted in bold.

**Fig. 13:**
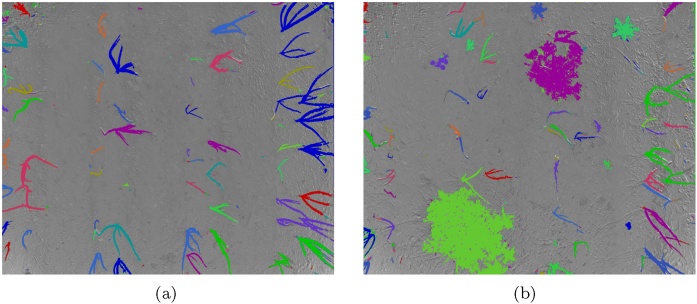
Segmentation results of onion images from Fig. 7 superimposed over the original RGB images, with each detected region coloured with a different colour. (For interpretation of the references to colour in this figure legend, the reader is referred to the web version of this article.)

The final output of the segmentation step is a list of potentially nested regions which can be directly processed without the need for component labelling. A segmentation output where the detected regions are coloured with random colours is shown in Fig. 13, together with an example of a nested region capturing a single plant in a more complex, bigger region, which is an additional advantage for classification.

### 6.2 Plant classification

**Fig. 14:**
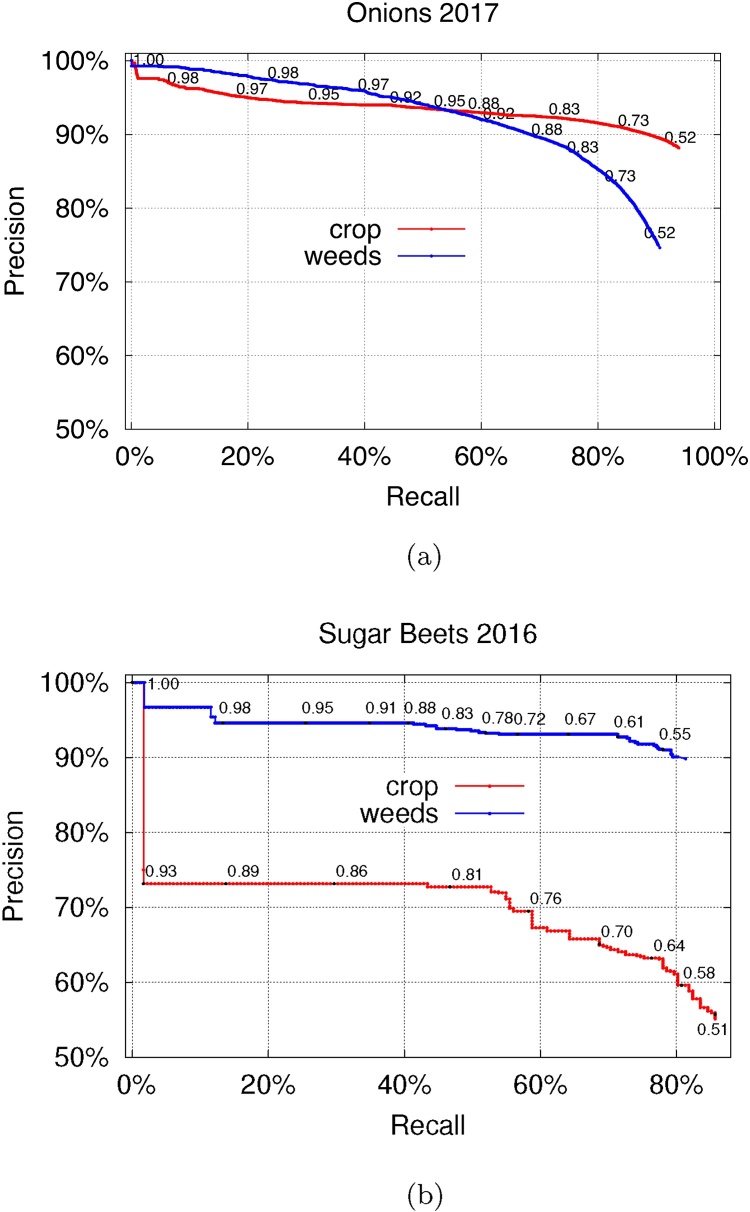
Precision-recall curves for classification between ‘crop’ and ‘weed’. The results on the *onions* images are shown in (a), and on the *sugar beets* images in (b).

The manually annotated subsets of the *Onions 2017* and *Sugar Beets 2016* datasets, detailed in Table 1, were used in the classification experiments. The corresponding precision-recall curves are shown in Fig. 14. The best *F*_1_ score achieved is 85% for the *Onions 2017* dataset, and 76% for the *Sugar Beets 2016* dataset. While our results on the sugar beets are not as good as the results reported by the authors of the dataset [[12], [6]], we note that we train our classifier on only 5 features per region as opposed to more than 300 in the original work.

**Fig. 15:**
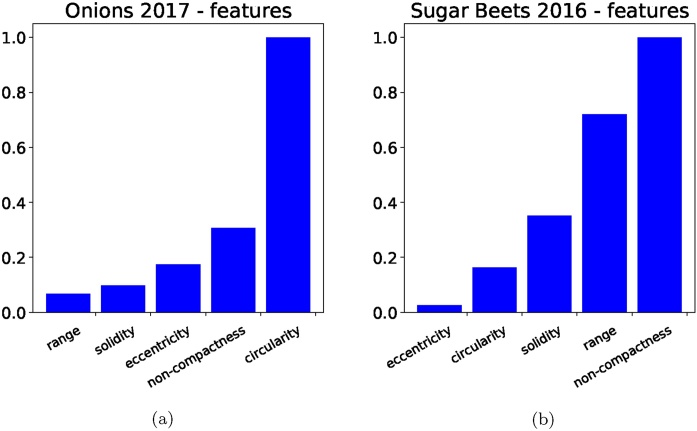
Feature importance based on the coefficients in the linear SVM classifier for the onions dataset is shown in (a) and for the sugar beets dataset in (b). We take the absolute value and normalise all the coefficients, making the weight of the most important feature 1.

**Fig. 16:**
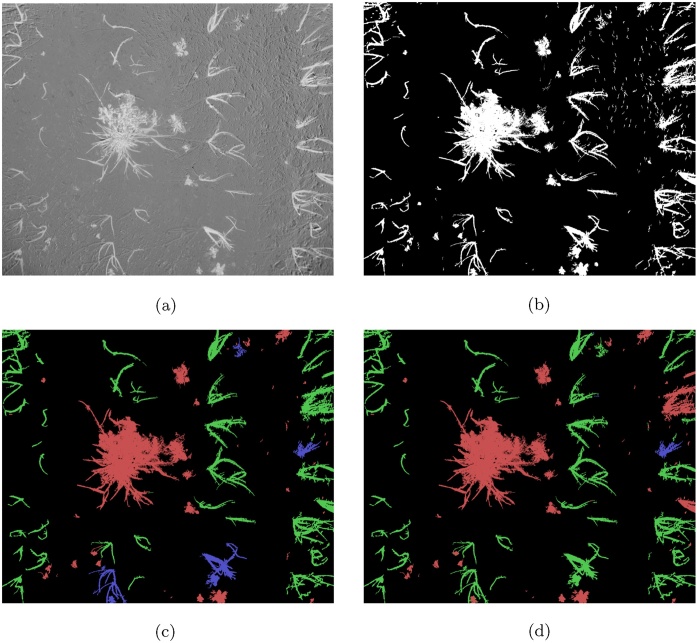
Example of vegetation region classification on an image from the *onions* dataset. The input NDVI image is shown in (a). The output of the segmentation step used for producing the ground truth data is shown in (b), and the resulting ground truth image is shown in (c) with crop regions marked in green, weed regions in red and mixed regions in blue. The output of the classifier is shown in (d). (For interpretation of the references to colour in this figure legend, the reader is referred to the web version of this article.)

**Fig. 17:**
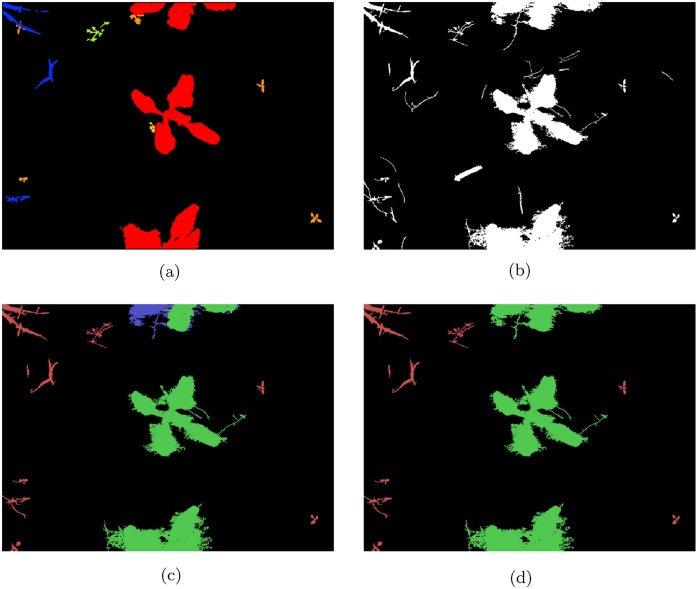
Example of vegetation region classification on an image from the *Sugar Beets 2016* dataset. The ground truth provided with the dataset is shown in (a). The output of the segmentation step used for producing the ground truth by labelling each region is shown in (b), and the resulting ground truth image is shown in (c) with crop regions marked in green, weed regions in red and mixed regions in blue. The segmented regions not corresponding to a vegetation region were discarded when manually producing the ground truth data. The output of the classifier is shown in (d). (For interpretation of the references to colour in this figure legend, the reader is referred to the web version of this article.)

We show the importance of the features based on the coefficient weights in the linear SVM in Fig. 15. Additionally, our classification results are based only on a small number of images from each of the used datasets. An example of manual classification and SVM output for the *Onions 2017* dataset is shown in Fig. 16, and similarly for the *Sugar Beets 2106* dataset in Fig. 17.

### 6.3 Complexity and performance speed analysis

From a theoretical point of view, we can analyse the complexity of the pipeline through these four steps:

(1) construction of the max-tree,
(2) calculation of leaf extinction values and maxima selection,
(3) region selection for segmentation,
(4) attribute calculation for feature description,
(5) region classification.

The max-tree construction (step 1) has been well-studied, and many algorithms of linear complexity in the number of image pixels were proposed [53] and successfully parallelised [33]. As with many other processing techniques in attribute morphology, both calculating the extinction values [48] (step 2) as well as attribute calculation (usually used for attribute filtering) [26] (step 4) have linear complexity in the number of image pixels, as they require one pass over the max-tree and can even be calculated simultaneously with tree construction. Since we use the SVM classifier in step 5, classifying a single sample corresponds to simply calculating a dot product and is thus very efficient. The theoretical complexity of step 3 is somewhat more difficult to express, as there is no guarantee on the number of maxima selected since we examine the whole branch for each selected maximum due to our adjustment process. While this can lead to high theoretical complexity (in the case of extremely degenerated trees, processing a whole branch for each leaf leads to quadratic complexity), in practice this is the fastest step of the algorithm, taking only about 10% of total processing time. When measuring the execution times of our segmentation algorithm on the sugar beets images on an Intel(R) Core(TM) i7-6700HQ CPU @ 2.60 GHz machine, we obtained an average processing time of 1.42 s/image (steps 1–3) for the segmentation, out of which the region selection took only 0.15 s/image (step 3). We also note that the pipeline is not optimised for speed in the current implementation, which can be improved by a full order of magnitude in more efficient implementations (e.g. [48]).

## 7 Conclusions and future work

We have presented a vegetation detection and classification pipeline for precision agriculture, which is fully based on various tools from attribute morphology applied to a single data structure, an image hierarchy called the max-tree. This image representation natively includes neighbourhood information, while processing it corresponds to working directly with regions present in the image and thus does not introduce any new edges or contours not present in the original image. The max-tree enables us to process the regions based on shape information as well as to process the image content locally.

The proposed pipeline was validated on two datasets, the *onions* dataset collected by the authors (including an artificially generated low-vegetation example) as well as the publicly available Sugar Beets 2016 [6] dataset. We present competitive results for both the segmentation and classification tasks. No isolated foreground noise is returned by the proposed max-tree segmentation algorithm, in contrast to both the classical Otsu's threshold selection [15] and the more local RATS approach [45], thus post-processing steps focussed on such noise removal can be avoided. Moreover, the goals of simple post-processing and noise-removal steps can be introduced directly as conditions, such as allowed region size, pixel intensity range or variation, to the pipeline without any increase in the algorithm complexity. We also demonstrate the robustness of the approach in low-vegetation situations. The output of the segmentation step is a set of (possibly nested) regions, which are then used directly for the classification process without the need for component labelling. While we acknowledge that there are some near-duplicates between these regions, there are cases where the sub-regions capture isolated plant structures that are parts of bigger regions, which are then further treated by the classifier. We also propose to use the region attributes calculated on the max-tree directly to produce feature vectors describing each region. Such feature vectors are then used as input to an SVM classifier, classifying the detected regions into ‘crop’, ‘weed’ and ‘mixed’ classes. We show good performance on both datasets used for validation with as few as 5 features, presented in terms of precision and recall.

Finally, we list several possibilities for improvement of the pipeline and future work. Reducing the number of minima processed in segmentation, which are currently determined based on their intensity extinction values, could significantly speed up the segmentation of vegetation. The selection criteria in segmentation should be further studied to better avoid near-duplicate regions while keeping the regions representing real sub-structures, and also in order to improve the response at the edge of the foreground regions. Additionally, this step can be redesigned so that it does not re-examine the same nodes in the hierarchy more than once (similar to MSER), in order to lower the high upper limit of the computational complexity of this step. Given the fact that the algorithm can natively include a parameter for the allowed region size, it would be interesting to calculate this once the precise camera and setup parameters are known in order to correlate it with the minimum physical dimensions of plants under observation. In order to improve the performance of the classifier, we intend to try out different classifiers on a larger dataset. We expect a further performance improvement by introducing more features to the region descriptor vectors, where it would also be interesting to try out traditional region or keypoint descriptors (e.g. SIFT [54], HOG [55], BRIEF [56]) or morphological ones (e.g. pattern spectra [29] or texture-based descriptors [57]). Lastly, we intend to construct a pixel-based classifier in order to deal with the ‘mixed’ class and, in general, cases too difficult to deal with for the region-based classifier. While such a classifier could be constructed based on geometrical, texture and shape features calculated on the local neighbourhood, the max-tree hierarchy could also be exploited to benefit from morphological pixel-based descriptors based on granulometries such as attribute profiles [58], which encode the behaviour of a certain attribute (size, shape or texture related) in the local neighbourhood of an image element.
